# The effectiveness of relaxation exercises on fatigue in hemodialysis patients: a meta-analysis study

**DOI:** 10.1590/1806-9282.20240680

**Published:** 2024-10-07

**Authors:** Ayşegül Yıldız İçigen, Emine Erdem

**Affiliations:** 1 Cappadocia University, School of Health Science, Department of Nursing – Nevşehir, Türkiye.; 2 Erciyes University, Faculty of Health Science, Department of Nursing – Kayseri, Türkiye.

**Keywords:** Exercise, Fatigue, Meta-analysis, Nursing, Renal dialysis

## Abstract

**OBJECTIVE:**

This study was conducted to determine the effect of relaxation exercise on fatigue symptoms in hemodialysis patients.

**METHODS:**

This is a meta-analysis study. The literature review was carried out by searching studies published between 2011 and 2020. This meta-analysis was recorded on PROSPERO in the National Health Research Institute (Registration no: CRD42022313646).

**RESULTS:**

Seven studies meeting the inclusion criteria were included in the meta-analysis. The effect size of the studies included in the meta-analysis was found to be g=1.232 (p=0.028), which indicated a "huge effect size." The scale used in the subgroup analyses, the application time of the relaxation exercise, and the number of relaxation exercise applications were evaluated, and a significant difference was found at p<0.05.

**CONCLUSION:**

Relaxation exercises can be used as an effective method for reducing fatigue in hemodialysis patients.

## INTRODUCTION

Although hemodialysis (HD) treatment, which is frequently and safely used in patients with end-stage renal disease, is beneficial for patients, it can also have negative effects on individuals’ lives^1^. Patients on hemodialysis treatment organize their lifestyles (work, social life, nutrition, meeting hygiene requirements, etc.) and all other activities according to dialysis treatment. Patients become dependent on HD centers, medical care, and medical personnel due to HD^2^. Negative changes caused by the disease and hemodialysis treatment cause patients to experience some problems over time. Fatigue, one of these problems, affects 60–97% of dialysis patients and is common^2–5^.

It is recommended to manage symptoms in HD patients experiencing fatigue by using pharmacological and non-pharmacological methods^6–10^. Relaxation exercises reduce muscle tension, stress, pain, anxiety, and fatigue, provide anger control, increase immunity, and improve sleep quality^10,11^. They are defined as non-invasive methods that are included in the nursing interventions classification are inexpensive, do not carry risks for sick individuals, and are easy to learn^12^. Also, they have a positive effect on mental health, pain, and fatigue and improve the quality of life in hemodialysis patients^11^. In addition to studies showing that relaxation exercises have a positive effect on relieving fatigue in hemodialysis patients^12,13^, there are also studies indicating that they do not make any difference and have no positive effect^14,15^.

With meta-analyses in the field of nursing, nurses can provide more effective and quality care, approach clinical problems with evidence-based problem-solving methods, keep nursing practices up-to-date, minimize errors, have a critical approach, and make effective decisions. For this reason, this meta-analysis study was conducted to examine the effect of relaxation exercises, which are easy to apply and require no cost, on fatigue in HD patients.

## METHODS

The PRISMA checklist was used during the conduct of this study. Research results were reported based on this checklist.

### Search strategy

The literature search was carried out online between January 2021 and July 2021 on the Cochrane Central Register of Controlled Trials, Web of Science, PubMed, Google Scholar, Ovid, and Science Direct databases. Also, the references of the included studies were used in the search. The titles, abstracts, and keyword lists of studies were searched by using the keyword combination "fatigue AND hemodialysis AND relaxation AND exercise."

### Eligibility criteria

The criteria for inclusion of studies in the meta-analysis during the literature review defined studies that were published between 2011 and 2020, included hemodialysis patients, were conducted in the field of nursing, were published in the English language, were randomized controlled or quasi-experimental, contained enough quantitative data, and had necessary data to calculate effect sizes and whose abstract or full text could be accessed.

### Risk of bias among studies

The random effects model was used to avoid the risk of bias in the meta-analysis. Publication bias was evaluated with the funnel plot.

### Statistical analysis

Statistical analyses were performed on the comprehensive meta-analysis software (version 3.0). The results were interpreted at a confidence interval (CI) of 95% and a p<0.05.

### Ethical aspects

Ethics committee approval was obtained from the local ethics committee before the research was initiated (decision no: 2020.30, date: 09.10.2020).

## RESULTS

### Quality assessment

Quality assessments of the studies included in the meta-analysis were carried out using the randomized controlled trials checklist and quasi-experimental studies checklist published by the Joanna Briggs Institute^16^. Evaluations were performed by three independent researchers [scoring of randomized controlled experimental studies: Cohen Kappa (k)=0.85; scoring of quasi-experimental studies: k=0.87].

### Search description

**Figure 1:**
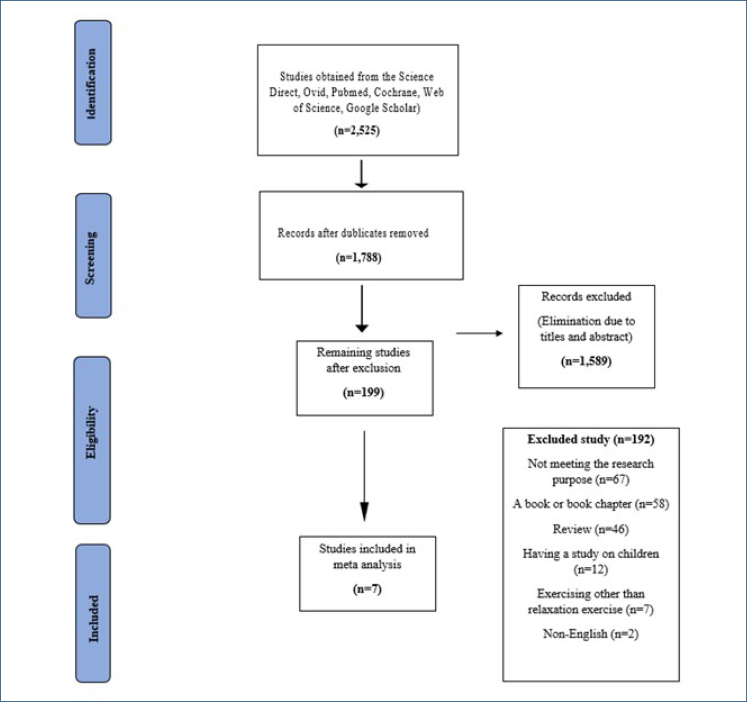
PRISMA flow chart.

The literature search yielded 2,525 studies, and 737 of them were excluded as they were duplicates. A PRISMA flowchart was used to report the meta-analysis (Figure 1).

### Characteristics of the studies included

The studies included in the meta-analysis were coded on a data collection form by using author-year, research model, sample group, duration of hemodialysis, and duration of relaxation exercise variables. The characteristics of the studies are listed in Table 1.

**Table 1:** Characteristics of studies ıncluded in meta-analysis.

Author (year) Country	Research model	Sample	Measuring tool	Frequency of hemodialysis	Relaxation exercise time
Kaplan et al. (2019)^11^ Türkiye	Randomized controlled trial	Experiment: 48 Control: 48	Piper Fatigue Scale	Three times a week	At least two times a week, for 6 weeks. The application lasted 20–30 min.
Hassanzadeh et al. (2018)^19^ Iranian	Randomized controlled trial	Relaxation: 35 Aromatherapy: 35 Control: 35	Brief Fatigue Inventory (BFI)	Three times a week	Two times a week for 4 weeks. The application lasted 20–30 min.
Hadadian et al. (2018)^14^ Iranian	Randomized controlled trial	Experiment: 27 Control: 38	Fatigue Check List (FCL)	Three times a week	It was applied twice a day for 4 weeks. The application took 15 min.
Amini et al. (2016)^17^ Iranian	Randomized controlled trial	Aerobic:32 Relaxation: 33 Control: 35	Piper Fatigue Scale	Three times a week	Two times a week; 30 min for 5 weeks.
Motedayen et al. (2014)^21^ Iranian	Quasi-experimental study	Experiment: 33 Control: 33	Fatigue Severity Scale (FSS)	Three times a week	Two times a week for 8 weeks. The application lasted 20 min.
Maniam et al. (2014)^20^ Malaysia	Quasi-experimental study	Experiment: 28 Control:27	Fatigue Scale (FACIT)	Three times a week	Three times a week for 12 weeks. The application lasted 30–40 min.
Hamed and Aziz (2020)^18^ Egypt	Quasi-experimental study	Experiment: 50 Control: 50	Fatigue Assessment Scale (FAS)	Three times a week	It was applied twice a day for 4 weeks. The application took 20 min.

### Heterogeneity

The heterogeneity level of the studies included in the study was evaluated with the Q statistics (Q=175.906) and the I² value (I²=96.589), and it was concluded that they did not show a homogeneous distribution (p=0.028).

### Synthesis of results

The effect size of the studies included in the meta-analysis was found to be g=1.232 (95%CI 0.132–2.331, p=0.028), which meant a "huge effect size" (p<0.05). This result of the study shows that relaxation exercises reduce fatigue in patients on hemodialysis (Table 2).

**Table 2:** Total effect size of studies ıncluded in meta-analysis.

Model type	Overall effect size						
	n	Hedge's g	Standard error	95% Confidence interval		Z	p
				Lower limit	Upper limit		
Fixed effects	7	1.049	0.103	0.847	1.251	10.197	0.000
Random effects	7	1.232	0.561	0.132	2.231	2.196	0.028

Z: Z value of effect size; Hedge's g: effect size; p: statistical significance level.

According to the results of the subgroup analysis performed according to the scale types, it was found that there was a significant difference between the scales used to determine the effect of relaxation exercise on fatigue (p<0.001). Considering the order of the effect level according to the average effect sizes of the scales used, it was seen that the "Fatigue Assessment Scale (FAS)" took the first place with g=4.362, which indicated a "huge effect size" (95%CI 3.643–5.081, p<0.001). When the average effect size of the number of relaxation exercises applied was evaluated, it was determined that 20 or more applications had a value of g=1.439 and a "huge effect size" (95%CI 0.801–2.078, p<0.001). When the average effect size of the relaxation exercise application times was evaluated, it was determined that a 30–40-min-long relaxation exercise application had a value of g=1.439 and a "very large effect size" (95%CI 0.801–2.078, p<0.001).

## DISCUSSION

The results of this meta-analysis yielded evidence that relaxation exercise had a "huge" effect on fatigue. Yang et al. evaluated the effect of progressive relaxation exercise on HD patients (n=688) in a meta-analysis of eight studies and stated that relaxation exercises significantly reduced fatigue and had a positive effect on HD patients^13^ (MD=-0.87, 95%CI −1.20 to −0.53, I^2^<50%, p<0.001). Song et al. conducted a meta-analysis study (n=139), which included three studies on the effect of exercise training on sleep quality, depression, and fatigue in HD patients and reported that exercise had a positive effect on fatigue^12^ (SMD=-0.85, 95%CI −1.2 to −0.5, I^2^=0%, p=0.81) (p<0.001).

One of the studies included in this meta-analysis, Hadadian et al., showed that the relaxation exercise intervention applied in HD patients had a negative effect with g=-1.472 and a huge effect level^14^ (95%CI −2.021 to −0.923, p<0.001). In this study, HD patients were given relaxation exercise training and were asked to practice this exercise at home for 15 min for 30 days. As a result of this study, in which patients were followed by home visits and telephone calls, it was found that the fatigue level of the experimental group increased after the intervention (p<0.05). Hadadian et al. found a negative effect on fatigue, which might have been because relaxation exercises had been performed under patient control. Since the exercise program had not been performed under supervision, whether it had been done at the right time and frequency and correctly had been limited to patients’ self-reports.

Many scales with different properties are used to measure fatigue. The FAS, FCL, FACIT, BFI, FSS scales, and Piper Fatigue Scale were used to measure fatigue in the studies included in this meta-analysis^14,17–21^. The properties of a measurement tool may affect the determination of post-intervention fatigue levels in experimental studies. As a result of the meta-analysis performed according to the scales used, it was determined that the average effect size (Qbetween) was "large" and that there was a significant difference according to the type of scale used (g=1.049, p<0.001). Based on these meta-analysis findings, it can be said that the use of the FAS may be more effective in evaluating fatigue in interventions performed with relaxation exercise to evaluate the effect of relaxation exercise more effectively.

Fatigue experienced by hemodialysis patients is a chronic problem. The FAS is used to evaluate chronic fatigue in terms of content and scope. The FAS has been frequently used in the literature to evaluate fatigue in patients with renal failure^22,23^. This scale has one dimension. The items on the scale focus on both the physical and mental fatigue levels of individuals. This feature of the scale may have enabled individuals to better define fatigue and use the scale more effectively.

In experimental studies conducted by applying relaxation exercises, there is no clear information about how many times the relaxation exercises have been performed in total during the study period. The total application number of relaxation exercises is different in studies conducted with HD patients^14,17–21^. The analysis results showed that performing relaxation exercises 20 times or more yielded a "huge effect size" (g=1.439, p<0.001). It can be said based on the results of this meta-analysis that more effective results are obtained when the number of relaxation exercise applications is 20 times or more to eliminate fatigue in HD patients.

For the relaxation exercise method to be effective and have an effect on some symptoms, it is recommended to be applied for a period of 3–6 months^24^. In studies included in this meta-analysis, it was determined that relaxation exercises were applied for 4–12 weeks, generally twice a week, and in some studies, it was applied twice a day^14,18^ (Table 1).

There is no definite information about how long relaxation exercises should be done to be effective. Different exercise durations were used in studies on the examination of the effect of relaxation exercise on fatigue^14,17,18,20,21^. Depending on the type of relaxation exercises, the application times may vary. It is recommended to do stretching-relaxation exercise for about 20 min, only relaxation exercise for about 10 min, and breathing relaxation exercise for about 3–4 min^25^. It is also recommended to do relaxation exercises for 15–20 min twice a day^24^. In this meta-analysis study, the subgroup analysis performed according to the application times of relaxation exercises indicated that the application times of the relaxation exercises were statistically significant according to the Qbetween value. As a result of the meta-analysis conducted according to the application time, it was determined that the average effect size was "very large" (g=1.312, p<0.001) and the most effective application time regarding the effect of relaxation exercise on fatigue was 30–40 min. Based on the findings of this meta-analysis study, it can be said that more effective results can be obtained when the application time of the relaxation exercise is 30–40 min to relieve fatigue in HD patients.

### Limitations

The limitations of this meta-analysis were that the number of studies in which relaxation exercises were applied to relieve fatigue in hemodialysis patients was inadequate; seven studies were included in the meta-analysis; only articles published in the English language were included in the meta-analysis; and only studies published between 2011 and 2020 were included.

## CONCLUSION

Relaxation exercises should be applied to HD patients at least 20 times or more and for 30–40 min in each session for the exercises to be more effective. The FAS should be used to evaluate fatigue. To achieve the management of fatigue in HD patients, relaxation exercises should be applied by nurses. The sample size should be more comprehensive. Multicenter studies should be planned.

## ETHICAL APPROVAL

This study was approved by the Cappadocia University Ethics Committee (Number: 2020.30, Date: 09.10.2020).
