# Erratum

**DOI:** 10.1590/1806-9282.20240680ERRATUM

**Published:** 2024-12-02

In the manuscript "The effectiveness of relaxation exercises on fatigue in hemodialysis patients: a meta-analysis study", https://doi.org/10.1590/1806-9282.20240680, published in the Rev Assoc Med Bras. 2024;70(10):e20240680, on page 1:

**Where it reads:**

ORIGINAL ARTICLE

**It should read:**

REVIEW ARTICLE

